# Implementation of novel and conventional outbreak control measures in managing COVID-19 outbreaks in a large UK prison

**DOI:** 10.1186/s12889-022-12991-7

**Published:** 2022-04-07

**Authors:** Paul C. Coleman, Adam Pailing, Anjana Roy, Éamonn O’Moore, Joht Singh Chandan, Victoria Lumby, Paul Newton, Anna Taylor, Esther Robinson, Jonathon Swindells, Sarah Dowle, Roger Gajraj

**Affiliations:** 1 Warwick Medical School, University of Warwick, Coventry, CV4 7HL UK; 2 Health Protection, United Kingdom Health Security Agency, Birmingham, UK; 3 National Health and Justice, United Kingdom Health Security Agency, Birmingham, UK; 4 Birmingham Medical School, University of Birmingham, Birmingham, UK; 5 Her Majesty’s Prison Service, Birmingham, UK; 6 Birmingham and Solihull Mental Health NHS Foundation Trust, Birmingham, UK; 7 National Infection Service, United Kingdom Health Security Agency, Birmingham, UK; 8 Black Country Pathology Services Department of Medical Microbiology, City Hospital, Birmingham, UK

## Abstract

**Background:**

Outbreak control measures during COVID-19 outbreaks in a large UK prison consisted of standard (e.g., self-isolation) and novel measures, including establishment of: (i) reverse cohorting units for accommodating new prison admissions; (ii) protective isolation unit for isolating symptomatic prisoners, and (iii) a shielding unit to protect medically vulnerable prisoners.

**Methods:**

Single-centre prospective longitudinal study (outbreak control study), implementing novel and traditional outbreak control measures to prevent a SARS-COV-2 outbreak. The prison held 977 prisoners and employed 910 staff at that start of the outbreak.

**Results:**

120 probable and 25 confirmed cases among prisoners and staff were recorded between March and June 2020 during the first outbreak. Over 50% of initial cases among prisoners were on the two wings associated with the index case.

During the second outbreak, 182 confirmed cases were recorded after probable reintroduction from a staff member. Widespread testing identified 145 asymptomatic prisoners, 16.9% of the total prisoner cases. The cohorting units prevented re-infection from new prison admissions and the shielding unit had no COVID-19 infections linked to either outbreak.

**Conclusions:**

Identifying and isolating infected prisoners, cohorting new admissions and shielding vulnerable individuals helped prevent uncontrollable spread of SARS-COV-2. These novel and cost-effective approaches can be implemented in correctional facilities globally.

## Background

Severe acute respiratory syndrome coronavirus 2 (SARS-CoV-2) was declared a public health emergency by the World Health Organisation (WHO) on 31st January 2020. After 1 year, SARS-CoV-2 had infected more than 100 million people globally, resulting in over 2 million deaths [1]. The focus of interest over this time has been the impact of COVID-19 outbreaks in healthcare, educational and community settings [2, 3], however, the propensity for explosive COVID-19 outbreaks is of particular concern within prisons and places of detention [4, 5].

Globally, prison populations are at increased risk of respiratory (e.g., COVID-19 and influenza) and non-respiratory (e.g., tuberculosis, hepatitis C and invasive group A streptococcal disease) outbreaks due to a combination of staffing challenges (high staff turnover and limited medical support and access to personal protective equipment), environmental issues (close confinement and poor ventilation) and host characteristics (high prevalence of comorbidities) [4, 6, 7], with COVID-19 incidence among prisoners reaching almost three times the level observed in the general population [8]. Indeed, a study at two Michigan based hospitals demonstrated that prisoner status was associated with a more severe COVID-19 clinical presentation, higher rates of intensive care unit admission and increased 30-day mortality [9]. Prison outbreaks can quickly overwhelm prison services and place increased pressure on surrounding healthcare and public health services. Consequently, the WHO has identified prisoners as a neglected population in terms of healthcare need exceeding resource availability [10].

Published figures available after the first year of the pandemic showed there had been 42,107 confirmed cases and 510 deaths amongst prisoners in the USA and 3460 confirmed cases and 73 deaths among prisoners/probation service users in the UK [11, 12]. While limited data for low- and middle-income countries (LMIC’s) make comparisons difficult [13], a 2020 review found COVID-19 outbreaks in African prisons to be lower than in high-income countries (HIC’s), at least during the first year of the pandemic. This was attributed to the large number of African prisons that engaged in releasing thousands of inmates into the community to reduce rates of overcrowding [13]. However, the burden that the COVID-19 pandemic has placed on healthcare systems in LMICs has far exceeded that observed in HIC’s [14], as a consequence of low rates of vaccine availability and herd immunity, as well long-term underinvestment in healthcare systems [15].

Outbreak guidance for prisons from the Centers for Disease Control and Prevention (CDC) [16] and WHO [17] emphasises the importance of hand-hygiene, social-distancing, isolation, ventilation, restricting admissions and rapid testing. However, these measures can be difficult to implement [18, 19], particularly against long-term under-investment in prison services [20]. Other novel and cost-effective approaches are therefore needed. A 2020 systematic review found the management of COVID-19 outbreaks within prison settings to be highly variable and not informed by public health guidance or the best available evidence [21]. The same review advocates for ‘*a public health approach to managing COVID-19 in prison*’ settings [21].

This is the first paper describing how a public health approach, based on UK guidance published early in the pandemic (April 2020) and introduced across the UK prison estate [22, 23] was effective in controlling the spread of SARS-CoV-2 in a large European prison (Prison A). Prison A is a category B (housing prisoners taken directly from courts) men’s prison in the UK. Prisons in the UK are categorised A to D based on prisoners’ risk of escape, harm to the public and threat to the control and stability of a prison, with category A housing prisoners that pose the most serious threat and category D providing minimal security. The public health approach outlined in this study consisted of the establishment of specific isolation / cohorting units, including (i) reverse cohorting units to accommodate new prison admissions; (ii) protective isolation units for isolating confirmed and suspected cases; and (iii) shielding units to protect the clinically vulnerable, in addition to traditional outbreak control measures.

## Methods

### Study design and study population

This is a single-centre prospective longitudinal study. The single centre is prison A, a men’s prison with a high turnover population and capacity for 977 prisoners. It is composed of eleven wings, a social care unit, separation unit and healthcare unit. Each wing accommodates 100–175 prisoners. Prison A employed 910 staff and was holding 950 prisoners at the start of the first outbreak.

The eleven wings comprise: A, B and C-wing (general population); D-wing (sex offenders); G-wing (enhanced prisoners); J-wing (older/more vulnerable); K- and L-wing (remanded and convicted prisoners); M-wing (integrated drug treatment service); and N- and P-wing (new arrivals).

### Case definitions

Definitions of possible and probable cases have changed over time. Definitions used in this report represent the definitions used by prison healthcare staff during the management of the outbreak [24]. Possible cases were individuals reporting symptoms consistent with an upper respiratory tract infection but without typical COVID-19 symptoms. Probable cases were individuals reporting one or more typical COVID-19 symptom, i.e., fever or temperature > 37.8 °C, new continuous cough or anosmia. Probable and possible cases receiving a negative PCR test result were still isolated and treated as possible / probable cases in case of false negative PCR test results, which are more frequent when disease prevalence is low and sensitivity is compromised. Confirmed cases were individuals with a positive SARS-CoV-2 test result.

### Outbreak control team

The Outbreak Control Team (OCT) was convened and chaired by Public Health England’s (PHE) local Health Protection Team (HPT) to coordinate investigation and management of the outbreak. Membership included (local and national) representatives from PHE, Prison A, NHS, local government, and Her Majesty’s Prison and Probation Service (HMPPS). Roles of the OCT included declaring the start and end of the outbreak. OCT recommendations were submitted to the appropriate organisation for consideration and implementation – for example recommendations that the prison should be closed to new admissions were submitted to HMPPS, since all population management decisions were taken at a national level.

### Cohorting and isolation units

A shielding unit was established on J-Wing on 6th March 2020 to safeguard 35 prisoners with chronic medical conditions. Enhanced levels of biosecurity (i.e., measures to prevent introduction of COVID-19) included controlled access via one entrance; full use of PPE by staff; appointment of dedicated prison/healthcare staff and unlocking (for showers, exercise and telephone access) a maximum of 11 prisoners at one time.

**Fig. 1:**
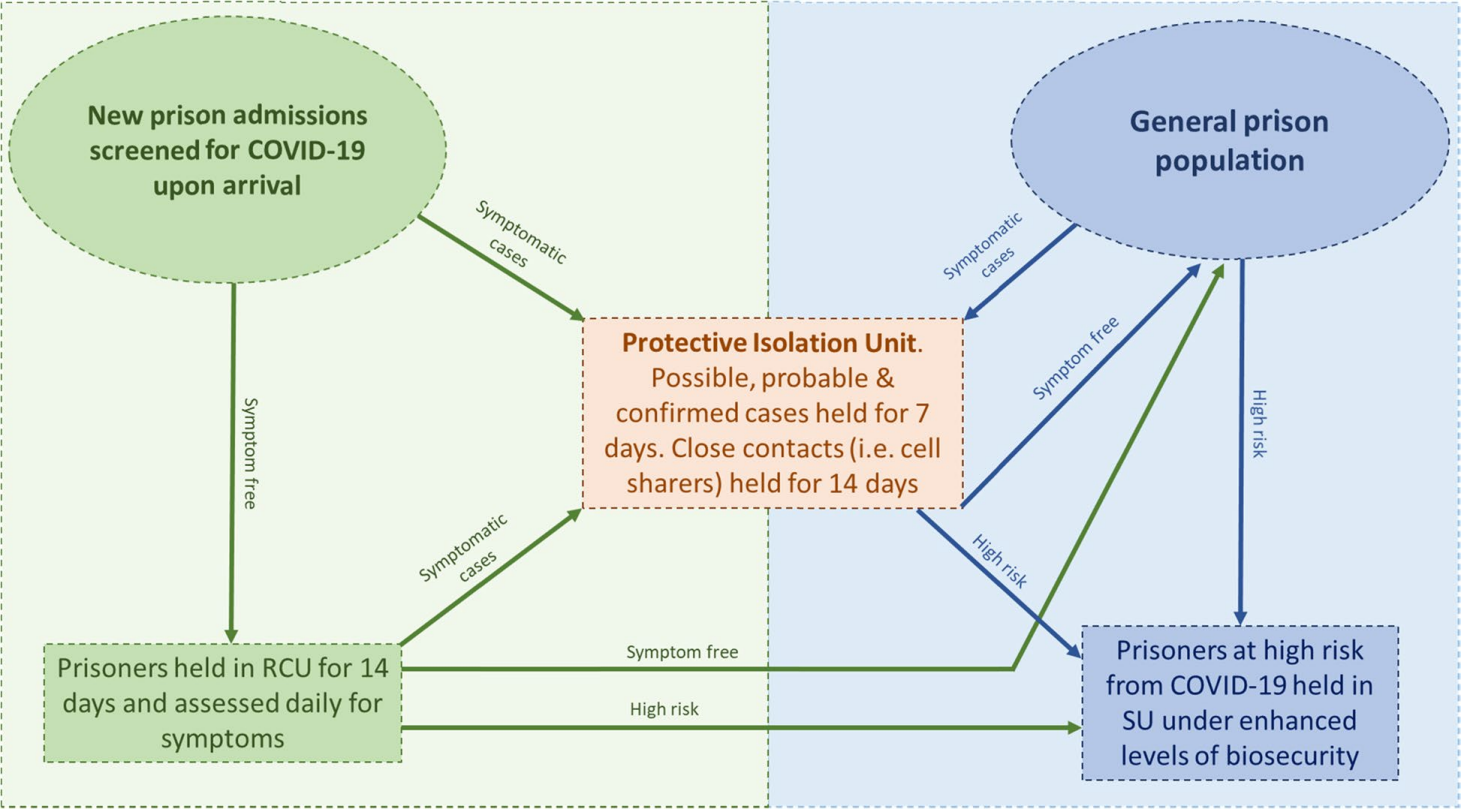
Outbreak control pathway for new admissions and the general prison population in Prison A. Key features of the outbreak response are the Reverse Cohorting Unit (RCU), Protective Isolation Unit and Shielding Unit (SU)

On the 10th April 2020 a reverse cohorting unit was established with the dual purpose of protecting the main prison population from imported infections, as well as protecting new admissions from any outbreak among the existing prison population. A protective isolation unit was established at the same time to isolate confirmed/suspected COVID-19 cases and close contacts for the duration of the infectious period (Fig. 1).

From 11th April 2020 onward, new arrivals underwent healthcare screening for COVID-19 symptoms and were either (i) quarantined in the reverse cohort unit for 14 days if symptom free, (ii) held in the protective isolation unit for 7 days if symptomatic or (iii) transferred to the shielding unit if identified as clinically vulnerable to COVID-19 (Fig. 1).

Shielding and cohorting units were established in non-central locations, away from the main population, with no thoroughfare and a single point of entry. Single cells were preferred, and facilities (showers, phones, exercise yard etc.) separated from those used by the main population. The Units had designated medical treatments rooms and access to a constant interaction cell (location used for prisoners deemed high risk of suicide). The reverse cohorting unit also had a dedicated area to conduct medical interviews and clinical assessments.

### Laboratory testing

In the early part of the study, only a minority of symptomatic prisoners were tested because of logistical difficulties in arranging testing undertaken by external healthcare staff. Once infection had been identified in the first cases, those in the prison with symptoms were assumed probable.

Arrangements were made for on-site swabbing, allowing all symptomatic prisoners to be rapidly tested, late in the first outbreak (12th May 2020). Prisoners had a nose *and* throat swab collected by a member of the prison healthcare team using the woven swab from a cobas® PCR Dual or Uni Swab collection kit. These were referred to a local NHS provider and tested using the cobas® SARS-CoV-2 dual target real time PCR assay (Roche Diagnostics, Switzerland). Staff sourced testing in the community via local NHS providers. During the second outbreak, samples from prisoners and most staff were routinely sent to the local Public Health England laboratory. Results were returned electronically to the prison healthcare team.

### Data analysis

Epidemic curves were constructed for staff and prisoners using date of onset of symptoms (symptomatic cases) or date of positive test result (asymptomatic cases). The date of implementation of outbreak control measures are demarcated in the epidemic curves. Demographic and clinical data and SARS-CoV-2 test results were abstracted from prison records by healthcare staff.

The attack rate (AR) was calculated for each prison wing using occupancy data from the start of each outbreak. Prisoner location is defined as the wing the prisoner was located on when symptoms first developed or immediately prior to being tested if asymptomatic.

## Results

### First outbreak

The first OCT meeting was convened on 23rd March 2020 following confirmation of a positive SARS-CoV-2 test result in a prisoner (index case with epidemiological links to G-wing and K-wing). The index case was admitted to hospital on 18th March 2020 with non-COVID-19 related symptoms, and discharged the following day. At the time of the first OCT meting there were an additional four prisoners and 40 staff isolating with probable COVID-19 symptoms. An outbreak was declared and prison lockdown implemented, including cessation of all admissions and transfers from courts and other prisons.

In total, 88 prisoners (*n* = 950, 9.2%) and 184 staff were identified as possible, probable or confirmed COVID-19 cases (Table 1). The outbreak was declared over at the OCT meeting on 26th June 2020 when there had been no confirmed cases among staff or prisoners for over 28 days.

**Table 1:** Demographic characteristics and COVID-19 diagnosis in prisoners and staff at Prison A

	First outbreak		Second outbreak	
	Prisoners (***N*** = 88; 9% of total prison population)	Staff (***N*** = 184; 20% of total staff population)	Prisoners (***N*** = 160; 19% of total prison population	Staff (***N*** = 22; 5% of total staff population
**Characteristics**				
Age - no. (%)				
20 - 29	26 (30)	32 (17)	50 (31)	0 (0)
30 - 39	25 (28)	23 (13)	46 (29)	0 (0)
40 - 49	19 (22)	37 (20)	29 (18)	0 (0)
50 - 59	5 (6)	34 (18)	8 (5)	0 (0)
60 - 69	3 (3)	3 (2)	0 (0)	0 (0)
70 - 79	1 (1)	0 (0)	0 (0)	0 (0)
80 - 89	0 (0)	0 (0)	0 (0)	0 (0)
90 - 99	1 (1)	0 (0)	1 (1)	0 (0)
Unknown	8 (9)	55 (30)	26 (16)	22 (100)
Sex - no. (%)				
Male	88 (100)	0 (0)	160 (100)	0 (0)
Female	0 (0)	0 (0)	0 (0)	0 (0)
Unknown	0 (0)	184 (100)	0 (0)	22 (100)
Hospitalised - no. (%)				
Yes	4 (5)	1 (0.5)	1 (1)	1 (5)
No	84 (95)	183 (99.5)	159 (99)	21 (95)
**COVID-19 diagnosis - no. (%)**				
*Confirmed COVID-19: Total*	3 (3)	22 (12)	160 (100)	22 (100)
COVID-19 confirmed: Asymptomatic	1 (1)	2 (1)	145 (91)	5 (23)
COVID-19 confirmed: Atypical symptoms	0 (0)	4 (2)	0 (0)	1 (5)
COVID-19 confirmed: Typical symptoms	2 (2)	16 (9)	15 (9)	16 (73)
*Probable COVID-19: Total*	79 (90)	79 (43)	0 (0)	0 (0)
Probable COVID-19: Not tested	54 (61)	61 (33)	0 (0)	0 (0)
Probable COVID-19: Tested negative	25 (28)	18 (10)	0 (0)	0 (0)
*Possible COVID-19: Total*	6 (7)	83 (45)	0 (0)	0 (0)
Possible COVID-19: Not tested	4 (5)	83 (45)	0 (0)	0 (0)
Possible COVID-19: Tested negative	2 (2)	0 (0)	0 (0)	0 (0)

Among prisoners, all three confirmed cases and 22 (35%) of the probable cases occurred in the 29-day period before the protective isolation and reverse cohorting units were established on 11th April 2020. Over the remainder of the outbreak there were zero confirmed cases and 23 probable cases among the resident population, in addition to 15 probable cases imported from the courts. There were no deaths and four prisoners required hospitalisation.

Seven of the nine wings reported probable or confirmed cases of COVID-19, with at least one case identified on each wing before the protective isolation and reverse cohorting units were established. Following their implementations on 11th April, the majority of new cases were among new admissions to the prison (15; 23.8%).

**Fig. 2:**
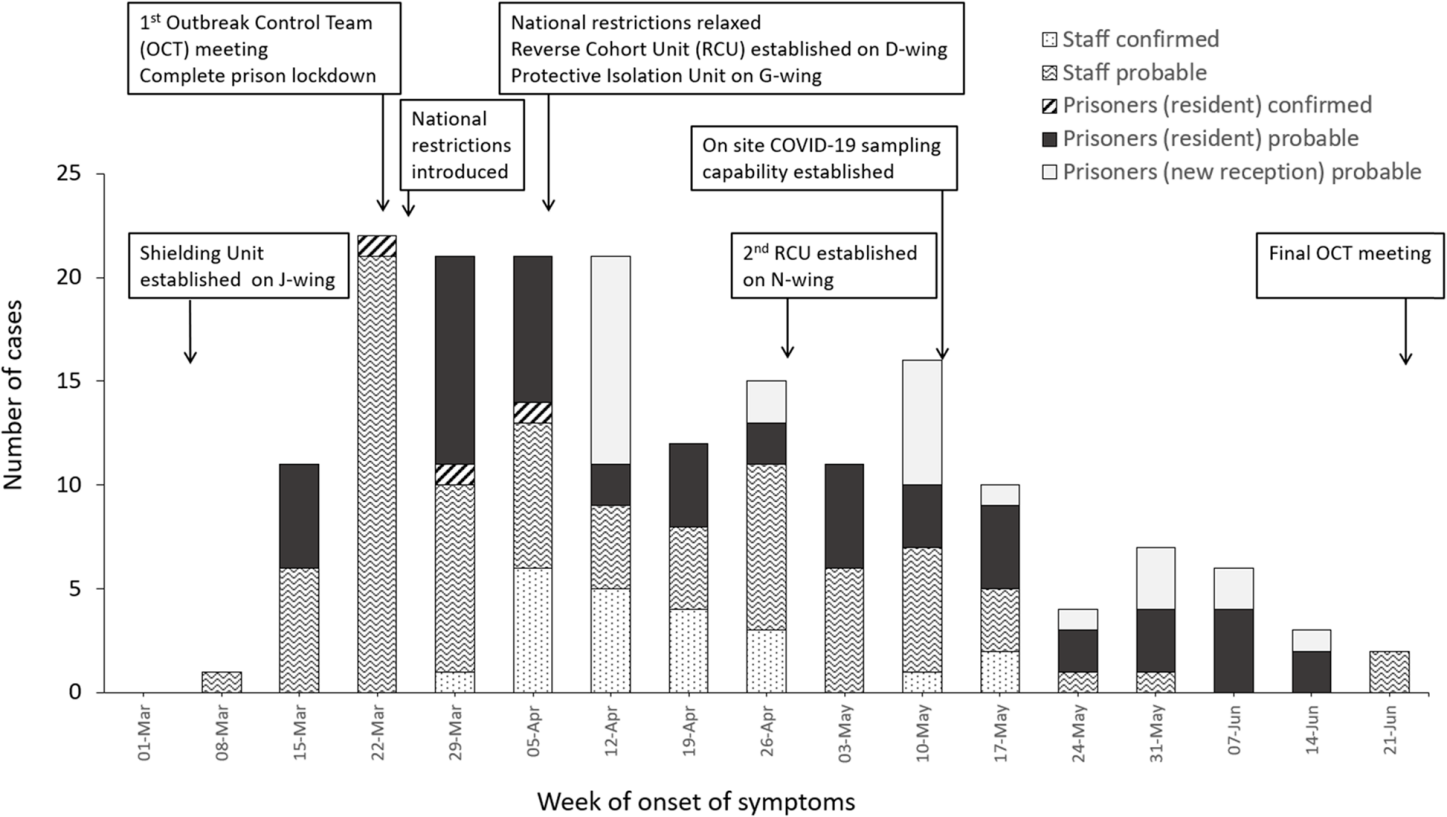
Probable and confirmed cases of COVID-19 by date of onset of symptoms among prisoners and staff linked to Prison A during the first outbreak and timeline of key events

Among staff, there were 83 possible cases, 79 probable cases, and 22 confirmed cases (Fig. 2). There were no deaths but one staff member required hospitalisation.

### Second outbreak

The OCT was re-convened on 20th November 2020 and a second outbreak was declared after Prison A reported six new cases, including one prisoner transferred to another prison, all with epidemiological links to M-wing. Subsequent investigation identified a seventh case in a member of staff who likely re-introduced infection on 12th November 2020 into Prison A onto M-wing. Mass testing of all prisoners and staff associated with M-wing was undertaken on 24th November: 113 (72.9%) out of 155 prisoners tested positive, of which 108 were asymptomatic. Of the 36 staff linked to M wing there were 9 positive results (25%).

Identification of further cases led to OCT recommendations for additional mass testing, first on the adjacent L-wing then subsequently whole prison testing. Six additional cases, all asymptomatic, were identified from 127 L-wing prisoners tested on 30th November 2020. Whole prison testing between 7th and 11th December identified 26 further prisoner cases and five further staff cases. Of particular concern were two cases on the shielding unit (J-wing) but investigations concluded that these were not linked to the prison outbreak – one was the carer (prisoner) of another medically vulnerable prisoner whose infection was likely hospital-acquired. There were no further cases among prisoners or staff on the shielding unit identified from mass testing on 20th and 30th November 2020.

**Fig. 3:**
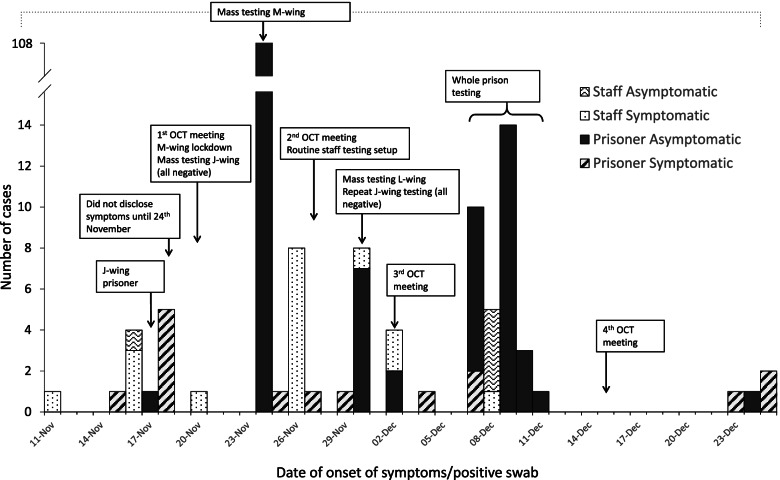
Confirmed cases of COVID-19 by date of onset of symptoms or date of positive swab for asymptomatic prisoners and staff linked to Prison A during the second outbreak and timeline of key events

In total, 160 confirmed cases were identified among prisoners during the second outbreak (18.7% of the total prison population) (Fig. 3 and Table 1). Prison testing identified COVID-19 cases in all wings except for H-wing, and P-wing. Among 460 staff, 22 positive cases (4.7%) were identified including four asymptomatic cases. The second outbreak was declared over on the 22nd January 2021 when there had been no new confirmed cases for 28 days.

## Discussion

### Statement of principal findings

This is the first publication to examine the public health management of a COVID-19 outbreak in a prison setting, and the first to examine the role of cohorting, isolating and shielding of prisoners to control the spread of COVID-19. In total, 120 probable and 25 confirmed COVID-19 cases among prisoners (9% of all prisoners) and staff (20% of all staff) were recorded over a 15-week period during the first outbreak. During the second outbreak, 182 confirmed cases among prisoners (19% of all prisoners) and staff (5% of all staff) were recorded over a 9-week period. We describe innovative arrangements used by Prison A to control the spread of COVID-19 through establishment of reverse cohorting units, protective isolation units and shielding units. These measures were implemented in addition to gold standard outbreak control measures such as social distancing, enhanced cleaning and use of face masks.

### Role of reverse cohorting, isolation and shielding units

Isolating and cohorting are well-established public health strategies for helping to control spread of infection and for protecting vulnerable individuals at increased risk of severe disease if infected [25]. But isolation of new prison receptions, and use of reverse cohorting units, is not routinely practiced in US prisons [16] nor part of current WHO recommendations [17]. We believe that more effective cohorting and isolation within prisons can be facilitated by the creation of dedicated facilities for this purpose. The reverse cohorting and isolation units should be separated from the main prisoner population in non-central locations with dedicated staff. As demonstrated during this outbreak, cohorting and isolating within physically distinct units can reduce the spread of infection across the prison, while affording better protection for uninfected new receptions and vulnerable residents. This might help explain the relatively low AR among prisoners reported during the first (9%) and second (19%) outbreaks, as well as in other UK prisons [26], in comparison to the AR of 80% reported for the Marion Correctional Institution in Ohio, USA [27].

Furthermore, the reverse cohorting unit acted as an effective buffer to prevent incursion of infection from new admissions, which are recognised as important sources of infectious disease [21, 28]. Standard guidance during outbreaks is for prisons to restrict new receptions, however, prison authorities often find this difficult to implement due to political pressure to receive transfers from courts and other prisons. However, as demonstrated by Prison A, the admission and release of prisoners can be continued, while controlling the spread of COVID-19, through the prompt establishment of reverse cohorting units.

COVID-19 transmission within any institutional setting poses a serious threat to medically vulnerable members of the population [3, 11], but the shielding unit was effective in preventing infection among this vulnerable cohort in both outbreaks. There were no cases in the shielding unit in the first outbreak. During the second outbreak there were only two linked cases on the shielding unit in a prisoner whose infection was likely hospital-acquired and his carer whom he likely infected.

The constant movement of staff members between the community and correctional facilities places them at high risk of introducing infection, as is documented in other infectious diseases [29, 30], and has been seen in staff transmission of COVID-19 in care homes in the UK [31, 32]. It is likely that prison staff played an important part as the source of infection in both outbreaks – in the first outbreak most of the initial cases were among staff (Fig. 2); in the second outbreak, a strong epidemiological association was established between the start of the outbreak on M-wing and a staff member working on that wing while displaying symptoms. Routine weekly testing of staff using PCR, then subsequently twice weekly lateral flow device (LFD) testing, was introduced towards the end of the second outbreak, but testing was voluntary and uptake was poor. Even if all symptomatic staff are excluded from work, data suggest that 20-50% of infections may be asymptomatic [33, 34] and that 40% of transmission occurs before symptoms develop [35, 36]. Studies of SARS-CoV-2 in prisons have thus far failed to regularly test prison staff or have tested staff on a voluntary basis [19, 37, 38]. More crude measures, such as temperature checks, also will not identify asymptomatic carriers [19], and one off screening has produced zero COVID-19 cases amongst prison staff [39].

While Prison A implemented national guidance that was replicated across the England, guidance itself only ever provides over-arching advice on how to control an outbreak. The innovation comes at a local level in responding creatively to the specific challenges of the outbreak, such as considering specific population needs and managing demand from across the wider justice system. The effective working between the prison, healthcare, public health and other partners, outlined in this manuscript, provides a role-model for prisons and places of detention globally.

### Unanswered questions and future research

Prisons are high-risk environments for outbreaks of infection partly because of the high population density, including medically vulnerable individuals, living in an enclosed space. Infection can be introduced to this semi-closed environment by staff or new prisoner admissions. Reverse cohorting units can effectively mitigate the risk presented by new prisoner admissions. But research is needed to identify the optimum strategy for preventing introduction of infection from staff.

The second outbreak was possibly caused by a member of staff continuing to work while symptomatic. There should be no financial disincentives if staff are justifiably required to self-isolate on multiple occasions because of symptoms, confirmed infection or contact with cases at work or in the community. In the second outbreak there was an explosion of cases on M-wing which were almost all asymptomatic, providing good evidence that asymptomatic cases spread infection, at least in a prison environment from that particular strain of COVID-19. More research is needed into which strains of COVID-19 are associated with a higher risk of asymptomatic transmission, and what testing strategy is most effective at identifying asymptomatic infectious cases. Population-level testing to identify asymptomatic cases using LFD testing with rapid results has been tried in the UK but questions have been raised about the cost-effectiveness of this policy and validity of the test [37]. Additionally, it has been suggested that asymptomatic cases are relatively uninfectious [38, 40].

Widespread COVID-19 screening of new admission to prison settings might not detect many cases [39]. However, findings from this study show that reverse cohorting units can prevent incursion of infection from the community into the prison. Testing of contact traced asymptomatic prisoners can also significantly reduce the spread of COVID-19 in prisons [19], and mass testing in an outbreak situation has the potential to identify a high number of asymptomatic cases [41]. In the second outbreak, the large number of cases on M-wing would not have been identified without mass testing. Better evidence regarding infectiousness of particular strains and the extent of transmission from asymptomatic and pre-symptomatic cases would also inform the testing strategy for outbreak investigation in prisons and other institutional settings.

To the best of our knowledge, this is the first publication to consider the role of specific infection control measures in controlling the spread of COVID-19 within a prison setting. Clearly there is an urgent need for additional research on control measures that are effective in controlling respiratory disease outbreaks in prisons and other places of detention.

### Implications for policy makers

For policy makers, the challenge is not simply to prevent the spread of COVID-19 infection within prisons, but to address the underlying cause of health inequities which result in prisoners being at higher risk of COVID-19 and other infections. As prisoners move from prisons to the community (and vice versa), any action to improve health outcomes within the prison environment will also improve health in the wider community [10]. It is essential that prisons are explicitly considered when preparing for - and responding to - pandemics and other health emergencies. The WHO has urged policy-makers to apply a ‘whole-of-government’ approach to improving the health of prisoners, through legislation to improve prison health; strengthening the interface between prison health systems and wider national health systems; and supporting evidence-based practice [10].

### Strengths and weaknesses of the study

There are a number of limitations associated with this outbreak report. Inadequate community and prisoner testing during the early stages of the first outbreak (only 33% of probable cases were tested) means there was likely an under-estimate of confirmed cases among prisoners and staff. This in part explains the low number of confirmed cases (three) among prisoners despite the large number (81; 92%) with classic symptoms. This is especially relevant given that testing was mostly unavailable in the early part of the first outbreak when outbreak incidence in the community was at its highest. A further limitation is that as Prison A introduced multiple interventions over a similar timeframe it is difficult to ascertain effectiveness of individual interventions. Due to the nature of the data collected it is also not possible to make inferences into behavioural/socio-economic risk factors associated with infection.

While there is generally limited risk of bias associated with outbreak control studies, there is potential risk of measurement bias in the identification of possible and probable cases, which were based on presentation of symptoms, during the first outbreak. However, widespread PCR testing of prisoners during the second outbreak reduced likelihood of measurement bias during later stages of the outbreak. There is also potential for a reverse healthy-worker effect, with members of the prison population at increased risk of COVID-19 due to increased presence of co-morbidities and close person-to-person contact, however, this does not affect generalisability in applying findings to other prisons and places of detention.

While outbreak control measures outlined in this manuscript can be implemented in diverse prison setting, limitations in external validity include the high turnover population of prison A increasing the likelihood of COVID-19 incursion into the prison. In addition, prisons in countries such as the USA and Africa have notably larger populations than UK prisons and may experience difficulty in allocating staff to establish cohorting, isolation and shielding units and in identifying adequate space for housing these specialist outbreak units. Prison regimes (i.e., legal requirements on the time prisoners spend outside of cells) also vary by country and may have an impact on holding prisoners in shielding and isolation units. Finally, characteristics of prison populations (e.g., gender, age, ethnicity and presence of co-morbidities) and physical characteristics of prisons (e.g., single, double and multi-occupancy cells) all vary within and between countries, all of which influence the ability to isolate and shield prisoners. However, despite these limitations, the evidence presented in this manuscript, documenting the progression of two COVID-19 outbreaks within the same prison, suggests that prompt establishment of reverse cohorting, protective isolation and shielding units can assist in preventing uncontrollable spread of SARS-COV-2.

## Conclusion

The risk of large outbreaks in prison settings will continue until universal vaccination of prisoners and staff is achieved. Well established control measures for outbreak management include isolation of cases and cohorting of staff to work in particular areas. These dedicated units provide not only more effective isolation and cohorting to help control spread of infection, but having the cohorts all located in one area also allow for more efficient monitoring and care provision by healthcare staff. These novel and cost-effective approaches can be implemented in correctional facilities globally.

## Data Availability

The datasets used and / or analysed during the current study are available from the corresponding author on reasonable request.

## Abbreviations

AR: Attack Rate
CDC: Centers for Disease Control and Prevention
HICs: High-income countries
HMPPS: Her Majesty’s Prison and Probation Service
HPT: Health Protection Team
LFD: Lateral flow device
LMIC’s: Low- and middle-income countries
OCT: Outbreak Control Team
PHE: Public Health England
SARS-CoV-2: Severe acute respiratory syndrome coronavirus 2
WHO: World Heath Organisation
